# Editorial: Transdetermination, transdifferentiation, and reprogramming of cells: *In vitro* and *in vivo* strategies

**DOI:** 10.3389/fmolb.2023.1194013

**Published:** 2023-04-13

**Authors:** Artur Cieslar-Pobuda, Essam M. Abdelalim, Shelley Bhattacharya

**Affiliations:** ^1^ Centre for Molecular Medicine Norway, University of Oslo, Oslo, Norway; ^2^ Diabetes Research Center (DRC), Qatar Biomedical Research Institute (QBRI), Hamad Bin Khalifa University (HBKU), Qatar Foundation (QF), Doha, Qatar; ^3^ College of Health and Life Sciences, Hamad Bin Khalifa University (HBKU), Qatar Foundation (QF), Doha, Qatar; ^4^ Environmental Toxicology Laboratory, Department of Zoology, Visva-Bharati (A Central University), Santiniketan, West Bengal, India

**Keywords:** cellular reprogramming, stem cell, transdifferentiation, iPSC (induced pluripotent stem cell), transdetermination

The basic aim of the Frontiers Research Topic was to stimulate interest in the use of stem cells in various chronic diseases. Conversion of one differentiated cell into another was considered as a method of replacement of diseased cells; these cell type transformations are known as metaplasia. However, transdifferentiation triggered a fierce controversy with respect to its clinical significance, and fundamental questions were raised on whether such a system of conversion of the cell type would really be able to improve the medical condition of the tissue concerned. It was also accepted that some types of metaplasia may lead to the development of cancer (Slack and Tosh, 2001; Tosh and Slack, 2002). The reason was that, in human histopathology, it is not unusual to locate foci of a different cell type different from the original.

Examples prevail in the form of bone in the soft connective tissue and squamous cells in the glandular epithelium (Willis, 1960); however, these conditions have been observed in the female reproductive system, where each organ has histologically different epithelium (Slack, 1985; Slack, 1986). Much later, Wagers and Weissman (2004) discovered that mere localization of different cell types in a particular tissue does not mean actual transdifferentiation; some were resulted by cell fusion, thereby incorporating genetic markers from the donor cells. There may have been reprogramming of bone marrow-derived cells to various other cell types, albeit at insignificant frequency. It may also be opined that reprogramming of bone marrow does not have the variety of cell repository required for regeneration of the rest of the body.

It has been reported by Weintraub (1993) that it is possible to reprogram a variety of tissue culture cell lines to a myogenic phenotype using the specific myogenic factor MyoD. Later, Zhou and Melton (2008) reprogrammed a differentiated cell type to another by overexpression of relevant transcription factors responsible for the commitment processes in normal development. As shown by Sun et al., the conversion of one type of cells to another—bovine ear fibroblasts into adipocyte-like cells—can also be chemically induced by using a combination of small molecules. Falk reported Barrett’s metaplasia where the lower end of the esophagus changes from a normal stratified squamous epithelium into a columnar epithelium, resembling the stomach and intestinal epithelium (Falk, 2002). However, some controversy exists about the cell of origin: it may be esophageal basal cells, or gland cells, or cells from the adjacent region of the stomach (Wang et al., 2011). In this medical scenario of reprogramming of differentiated cells *in situ* by interfering with the normal path of development, it may be proposed that adult differentiated tissues retain certain amount of stemness, as evidenced in the adult rats (Sarkar et al., 2019).

Ulcerative colitis (UC) is an example of chronic inflammatory disease with mucosal injury being a significant issue. LGR5^+^ intestinal stem cells are important in repairing this damage and are regulated by the Wnt/β-catenin and Notch signaling pathways. As described by Zheng et al., understanding the molecular mechanisms regulating intestinal epithelial tissue renewal and regeneration can lead to development of clinical therapies for patients with UC.

In recent years, advanced bioinformatic tools have facilitated studies on efficient conversion of one cell type to another. In 2016, Rackham et al. (2016) presented a predictive system that compares gene expression data with regulatory network information to predict the most crucial reprogramming factors necessary for successful cell conversion. Since then, such tools have been evolving and now they enable us to produce almost any target human cell type from any source cell type by deploying epigenetic- and next-generation sequencing data.

Finding the right transcription factors, small molecules, and optimal culture conditions is only a part of the success of the cell conversion process. Another important aspect, where single-cell RNA sequencing and computational analysis are found extremely useful, is to precisely characterize cells and identify the origins of heterogeneity in cellular populations Ge et al., (Francesconi et al., 2019). Pihlström et al. presented a multi-omics approach to validate the conversion of human dermal fibroblasts into osteoblast-like cells. To provide an in-depth insight into the developmental processes, a sequential transcriptomic, proteomic, and phospho-proteomic profiling of transdifferentiating fibroblasts over time was introduced. All these advances in the field make the transdifferentiation process safer and one step closer to potential clinical studies.

In conclusion, transdifferentiation seems to be a promising alternative for reprogramming somatic cells into induced pluripotent stem cells (iPSCs) as it can minimize the risk of malignant transformation. Conversion of one mature cell type to other also presents a unique opportunity to develop a new class of therapies, through the delivery of the optimal combination of regulatory factors directly in the human body. Such *in vivo* processes would replace or repair cells that lost their primary functions as a consequence of disease and would bring them to a healthy state. Until such a scenario is possible, more basic research is needed to better understand mechanisms involved in cell conversion processes and associated risks. Safety-related questions are the priorities that await urgent answers through thorough, systematic studies.
